# Plant organ- and growth stage-diversity of endophytic bacteria with potential as biofertilisers isolated from wheat (*Triticum aestivum* L.)

**DOI:** 10.1186/s12870-022-03615-8

**Published:** 2022-06-06

**Authors:** Fahu Pang, Aili Tao, Camilo Ayra-Pardo, Tan Wang, Ziwei Yu, Siliang Huang

**Affiliations:** School of Life Sciences and Agricultural Engineering, Nanyang Normal University, Nanyang, 473061 Henan People’s Republic of China

**Keywords:** Plant-growth-promoting bacteria, Crop nutrition, Pot trial

## Abstract

**Background:**

Chemical fertilisers are extensively used for crop production, which may cause soil deterioration and water pollution. Endophytic bacteria with plant-growth-promoting (PGP) activities may provide a solution to sustainably improve crop yields, including in-demand staples such as wheat. However, the diversity of the PGP endophytic bacteria in wheat across plant organs and growth stages has not been thoroughly characterised.

**Results:**

Here, we report the isolation of endophytic bacteria from root, stem, leaf and seed of three winter wheat varieties at tillering, jointing, heading and seed-filling growth stages that were identified via 16S rRNA gene sequence analysis. Strains were screened for indole-3-acetic acid (IAA) production, potassium and phosphate solubilisation and the ability to grow on a nitrogen-free medium. Strain's capacity to stimulate various plant growth parameters, such as dry root weight, dry above-ground parts weight and plant height, was evaluated in pot trials. A total of 127 strains were randomly selected from 610 isolated endophytic bacterial cultures, representing ten genera and 22 taxa. Some taxa were organ-specific; others were growth-stage-specific. *Bacillus aryabhattai*, *B. stratosphericus*, *Leclercia adecarboxylata* and *Pseudomonas oryzihabitans* were detected as wheat endophytes for the first time. The IAA production, inorganic phosphorous solubilisation, organic phosphorus solubilisation, potassium solubilisation and growth on N-free medium were detected in 45%, 29%, 37%, 2.4% and 37.8% of the 127 strains, respectively. In pot trials, each strain showed variable effects on inoculated wheat plants regarding the evaluated growth parameters.

**Conclusions:**

Wheat endophytic bacteria showed organ- and growth-stage diversity, which may reflect their adaptations to different plant tissues and seasonal variations, and differed in their PGP abilities. *Bacillus* was the most predominant bacterial taxa isolated from winter wheat plants. Our study confirmed wheat root as the best reservoir for screening endophytic bacteria with potential as biofertilisers.

**Supplementary Information:**

The online version contains supplementary material available at 10.1186/s12870-022-03615-8.

## Background

Virtually all plants are hosts to endophytic microbes (usually bacteria and fungi) [1–3]. These microbial entities live within plant living tissues without damaging the host or eliciting plant disease symptoms [4, 5]. Furthermore, endophytic bacteria have many biological characteristics that are beneficial for their host plants, such as nitrogen fixation [6, 7], phosphate solubilisation [8, 9], and production of indole-3-acetic acid (IAA) [10, 11] and antimicrobial substances [12, 13], which can thus promote the growth of host plants and increase their resistance to biotic/abiotic stress [14, 15]. Based on the above attributes, endophytic bacteria with plant-growth-promoting (PGP) activities could be considered one of the most effective ways to reduce pollution associated with chemical fertiliser loads in agriculture for sustainable and environmentally friendly crop production.

Endophytic bacteria have been isolated and identified from many crops, including wheat (*Triticum aestivum* L.) [16], cotton (*Gossypium* spp.) [17], tobacco (*Nicotiana tabacum* L.) [18], sweet potato (*Ipomoea batatas* L.) [19], sugar beet (*Beta vulgaris* L.) [20], rice (*Oryza sativa* L.) [21] and maize (*Zea mays* L.) [22]. In some species, the composition of endophytic bacterial communities has been investigated across plant organs and growth stages. Using fluorescence in situ hybridisation (FISH) and microbial cultivation, Compant et al. (2011) have revealed that some particular bacterial taxa could only be isolated from the reproductive organs of grapevine (*Vitis vinifera* L.) [23]. Chen et al. (2014) -working with ginger (*Zingiber officinale* R.)- could isolate the highest number of bacterial taxa from the seedling than from any other stage of plant growth [13]. Jin et al. (2014) reported that the habitat (rhizosphere vs endosphere) and organ (leaf, stem and root) determined the bacterial community associated with *Stellera chamaejasme* L. [24]. These authors found similar bacterial profiles between rhizosphere and plant root and between leaf and stem. In noni (*Morinda citrifolia* L.), endophytic microbial communities have shown a considerable diversity across plant parts (i.e. root, branch, leaf, seed, and fruit) [25].

Previous research on wheat endophytes has been focused on the potential application of isolated strains in the biocontrol of economically important crop diseases [26–30], while fewer studies have investigated the microbial biodiversity in plant organs, particularly the root [31, 32], leaf [16], and seed [33]. Larran et al. (2002) reported the isolation of three bacterial strains characterised as *Bacillus* sp. and 130 fungal isolates -of which 19 fungal species were identified- from healthy leaves of three wheat cultivars at three plant growth stages (i.e. second node detectable, medium milk and soft dough stages) [16]. Robinson et al. (2016) reported that wheat bacterial endophyte communities were most abundant and heterogenous in roots compared to leaves, and the composition was influenced by the use of fertiliser and sample time [34]. Similar results were obtained by Gdanetz and Trail (2017) when they surveyed the wheat microbiome across plant organs and land management strategies, using high throughput sequencing techniques [35]. Recently, twelve different endophytic bacteria isolated from grains of spring wheat varieties grown in soils with low bioavailability of microelements were found to synthesise indole-related compounds, including IAA, with phytohormonal activity [36]. Here, we aimed to investigate the distribution of endophytic bacteria with PGP abilities in four plant organs (root, stem, leaf, and seed) of three winter wheat varieties and at tillering, jointing, heading and seed-filling stages of the plant growth. Plants were sampled in two provinces of China during 2012–2013. The isolated bacteria were assessed for IAA production, phosphate and potassium solubilisation, in vitro growth on a nitrogen-free medium and wheat growth promotion in soil with N-P-K macronutrients at low or suboptimal rates. We found the distribution of PGP endophytic bacteria in winter wheat plants was highly influenced by the plant organ and growth stage. Pot trials revealed several PGP strains with potential as biofertilisers for sustainable wheat production in China.

## Results

### Isolation and identification of endophytic bacteria from wheat

**Fig. 1:**
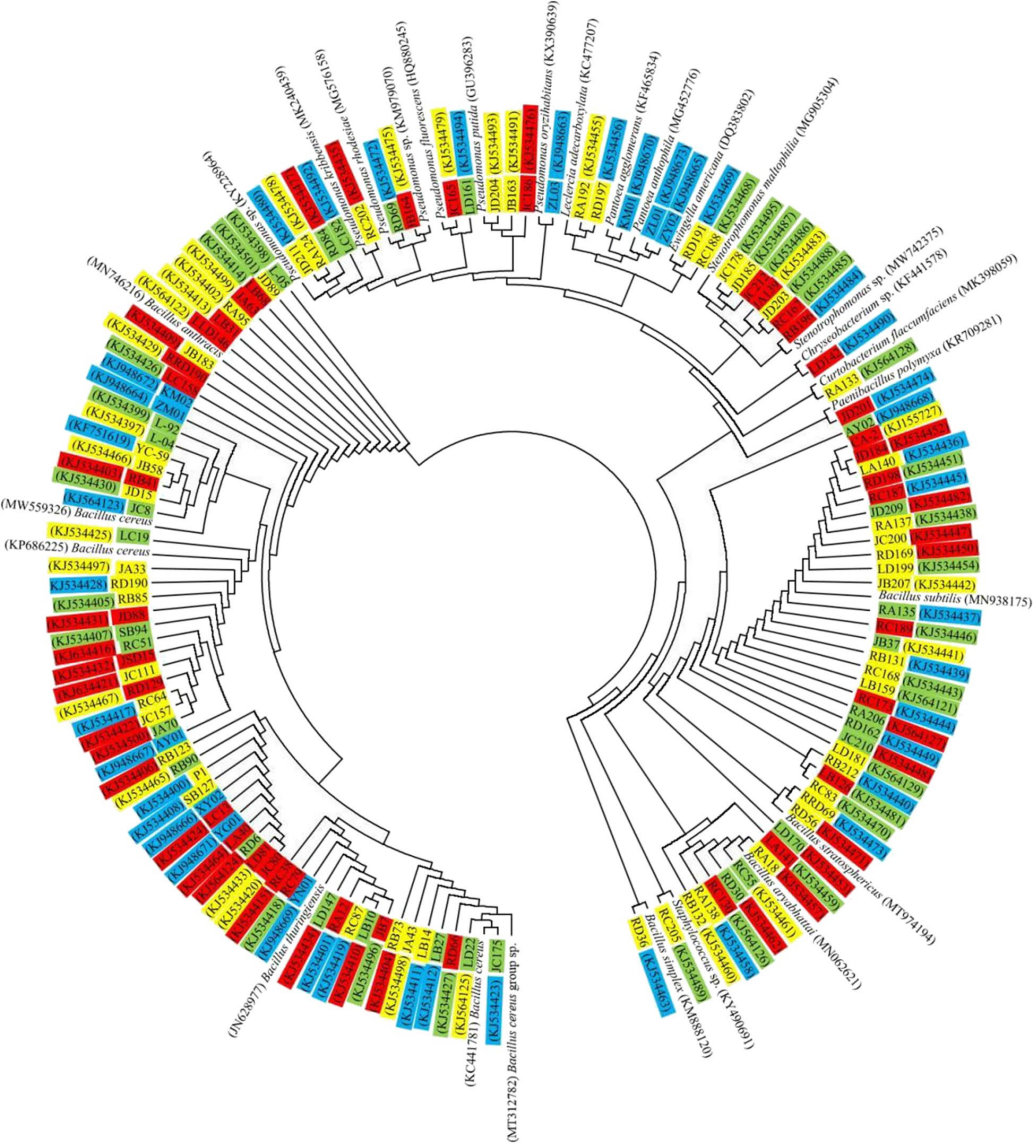
Neighbor-joining circle-shaped phylogenetic tree of 16S rRNA gene sequences showing the genetic relationship between the endophytic bacterial strains isolated from winter wheat plants and other related bacteria retrieved from GenBank database. The numbers in parentheses represent the accession numbers in GenBank. In the circular tree, coloured shades of the bacterial strain designations highlight the source of isolation among tillering (yellow), jointing (red), heading (green) or seed-filling (blue) stages. Similarly, coloured shades of GenBank accession numbers of bacterial strains distinguish the source of isolation among the root (yellow), stem (red), leaf (green), or seed (blue)

A total of 610 endophytic bacterial cultures were isolated from different organs (200 from the root, 180 from the stem, 140 from the leaf and 90 from the seed) of three winter wheat cultivars (Pumai 9, Bainong 207 and Jinmai 92) at the four growth stages (tillering, jointing, heading and seed-filling) following a culture-dependent protocol. Of the isolated bacterial cultures, 127 strains (51 from the root, 37 from the stem, 28 from the leaf and 11 from the seed) were randomly selected for further biochemical and molecular characterisation. The 127 strains were grouped into ten genera (*Bacillus*, *Chryseobacterium*, *Curtobacterium*, *Leclercia*, *Ewingella, Paenibacillus*, *Pantoea*, *Pseudomonas*, *Staphylococcus* and *Stenotrophomonas*) and further identified to be members of sixteen species (*Bacillus aryabhattai*, *B. stratosphericus, B. simplex*, *B. subtilis*, *Curtobacterium flaccumfaciens*, *Ewingella americana*, *Leclercia adecarboxylata*, *Paenibacillus polymyxa*, *Pantoea agglomerans*, *Pan*. *anthophila*, *Pseudomonas fluorescens*, *Ps. kribbensis*, *Ps. oryzihabitans*, *Ps. putida*, *Ps. rhodesiae*, and *Stenotrophomonas maltophilia*) and six unspecified taxa (*B. cereus* group sp., *Bacillus* sp., *Chryseobacterium* sp., *Pseudomonas* sp., *Stenotrophomonas* sp. and *Staphylococcus* sp.) (Fig. 1). *Bacillus* was found as the most predominant genus, followed by *Pseudomonas*, with 96 and 13 strains isolated, respectively. The results revealed considerable diversity in the endophytic bacterial community in wheat plants.

### Diversity of endophytic bacteria among wheat organs

**Fig. 2:**
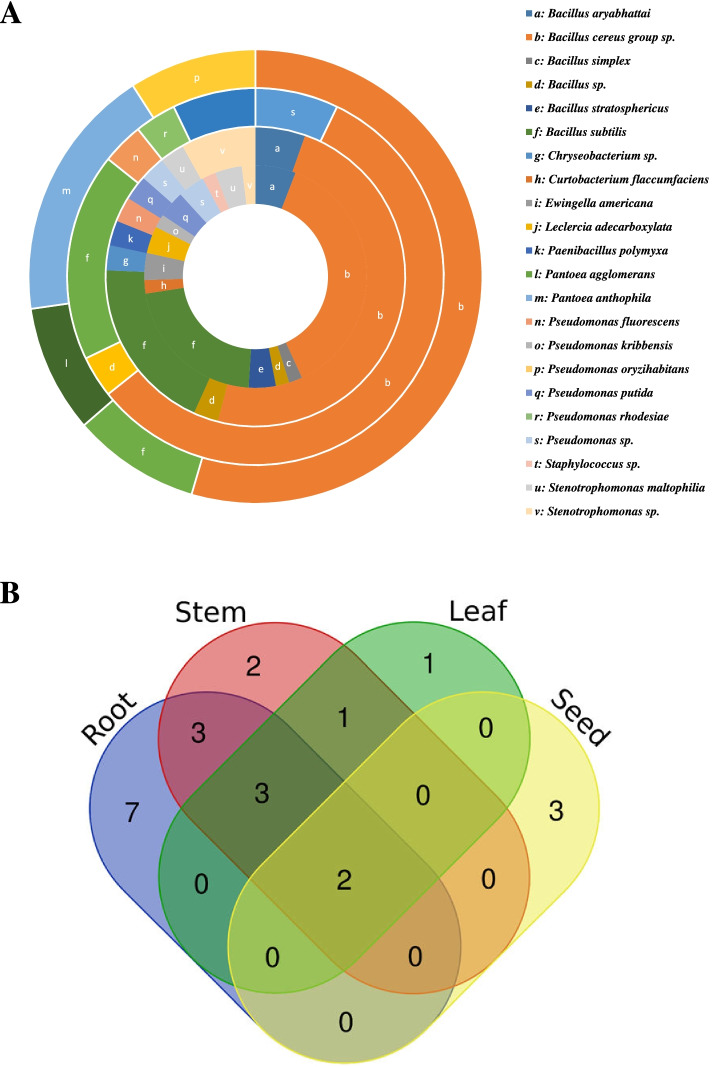
Isolation of endophytic bacteria from different plant organs of winter wheat plants. (A) The concentric rings in the doughnut chart display, from the inside to the outside, the proportions of the endophytic bacterial taxa isolated from the root, stem, leaf and seed, respectively. (B) A Venn diagram showing the distribution of the 22 bacterial taxa among the different wheat plant organs. The online tool used to calculate and draw the Venn diagram was from http://bioinformatics.psb.ugent.be/webtools/Venn/

The isolation frequencies of wheat endophytic bacterial taxa varied significantly from one plant organ to another (Fig. 2A). Notably, *B. cereus* group sp. had the largest proportions compared to the other taxa in the root, stem, leaf, and seed with 37.3%, 48.6%, 57.1% and 54.6%, respectively. The wheat root showed more diverse and abundant bacterial communities (16 taxa), with significantly highest Shannon and Simpson diversity indexes, followed by the stem and the leaf and seed (Table 1). According to the Species Evenness index, the bacterial communities in the seed were the most even (*P* ≤ 0.05). The distribution of the 22 bacterial taxa among the different wheat organs was further investigated using a Venn diagram (Fig. 2B). Thirteen taxa showed an organ-specific distribution, of which seven (*B. simplex*, *B. stratosphericus*, *Staphylococcus* sp., *Cu. flaccumfaciens*, *E. americana*, *L. adecarboxylata* and *Ps. kribbensis*) were found exclusively in the root, two (*Chryseobacterium* sp. and *Pae. polymyxa*) in the stem, one (*Ps. rhodesiae*) in the leaf, and three (*Pan. agglomerans*, *Pan. anthophila* and *Ps. oryzihabitans*) in the seed. While *B. cereus* group sp. and *B. subtilis* were isolated from all wheat organs, *Stenotrophomonas* sp., *St. maltophilia*, and *Ps. putida* were found only in the root and stem. *Bacillus* sp., *B. aryabhattai* and *Pseudomonas* sp. were isolated from the root, stem and leaf; *Ps. fluorescence* was isolated from the stem and leaf.

**Table 1:** Distribution and diversity of endophytic bacteria in different organs of wheat plants at four growth stages^1^

Wheat organ	Growth stage	Number of taxa detected	Shannon index	Evenness
root	Tillering	7	1.767 b	0.908 a
	Jointing	4	1.148 d	0.828 ab
	Heading	8	1.935 a	0.931 a
	Seed-filling	8	1.705 b	0.820 ab
stem	Tillering	4	0.939 de	0.671 c
	Jointing	3	0.796 e	0.724 b
	Heading	6	1.782 b	0.884 ab
	Seed-filling	6	1.549 bc	0.864 ab
leaf	Tillering	3	0.867 e	0.789 b
	Jointing	5	0.966 de	0.601 c
	Heading	1	0 f	0 d
	Seed-filling	5	1.494 c	0.928 a

^1^Different letters in the same column indicate significant differences at *P* ≤ 0.05 (ANOVA with Duncan's post-test)

### Diversity of endophytic bacteria among wheat growth stages

**Fig. 3:**
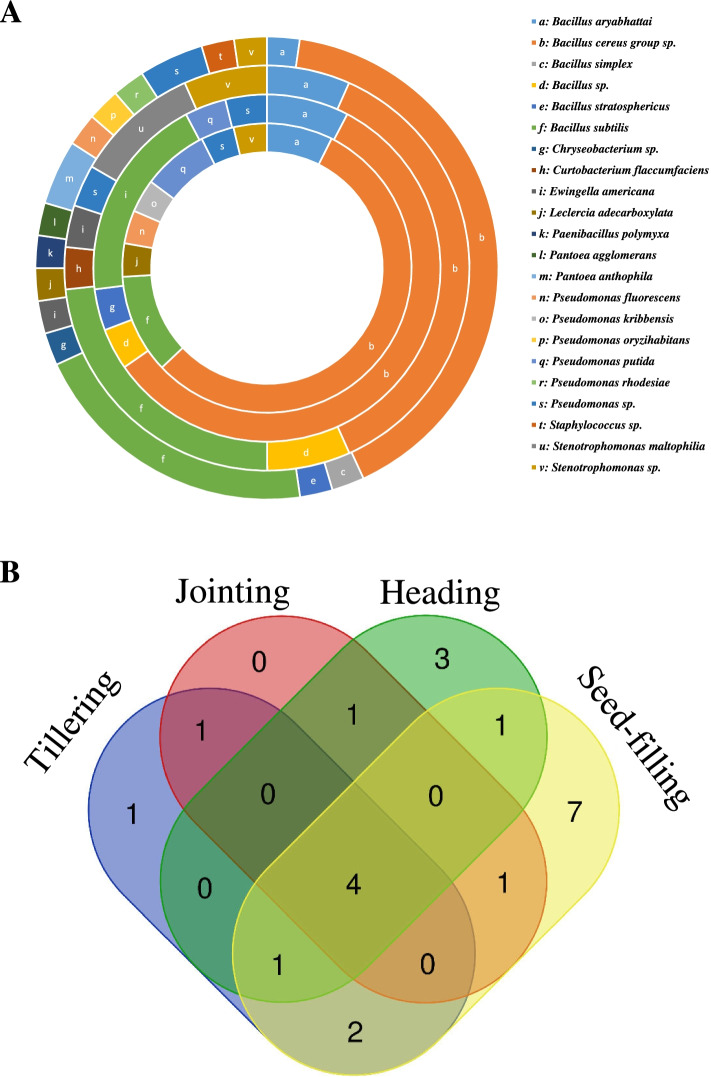
Isolation of endophytic bacteria at different growth stages of winter wheat plants. (A) The concentric rings in the doughnut chart display, from the inside to the outside, the proportions of the endophytic bacterial taxa isolated at the tillering, jointing, heading and seed-filling stages, respectively**.** (B) A Venn diagram showing the distribution of the 22 bacterial taxa among the different wheat growth stages. The online tool used to calculate and draw the Venn diagram was from http://bioinformatics.psb.ugent.be/webtools/Venn/

The isolation frequencies of wheat endophytic bacterial taxa varied significantly with the growth stage of wheat plants (Fig. 3A and B). *B. aryabhattai, B. cereus* group sp., *B. subtilis* and *Pseudomonas* sp. were found at all wheat growth stages (tillering, jointing, heading, and seed-filling). *Stentrophomonas* sp. was isolated at all growth stages except the jointing stage. *Bacillus* sp. was isolated at jointing and heading stages; *B. stratosphericus* was isolated at jointing and seed-filling stages; *E. americana* was isolated at heading and seed-filling stages; *L. adecarboxylata* and *Ps. fluorescens* were isolated at tillering and seed-filling stages; *Ps. putida* was isolated at tillering and jointing stages. Growth-stage-specific presence of endophytic bacteria was detected in a few taxa. *Curtobacterium flaccumfaciens*, *Staphylococcus* sp. and *St. maltophilia* were isolated only at the heading stage; *B. simplex*, *Chryseobacterium* sp., *Pae. polymyxa*, *Pan. agglomerans*, *Pan. anthophila*, *Ps. oryzihabitans* and *Ps. rhodesiae* were isolated only at the seed-filling stage; *Ps. kribbensis* was isolated only at the tillering stage. The number of isolated bacterial taxa increased after the jointing stage and reached their peak at the seed-filling stage.

### IAA production by endophytic bacterial strains

Sixteen out of 22 endophytic bacterial taxa produced IAA at various levels (Table 2), only *B. simplex*, *B.stratosphericus*, *Chryseobacterium* sp., *Cu. flaccumfaciens*, *Ps. kribbensis*, and *Ps. rhodesiae* showed no IAA production. The 58 IAA-producing strains represented 39.4% of the 127 endophytic bacteria tested. The IAA production varied significantly from one strain to another, even among members of the same species. The number of strains with a high-level (≥ 50 mg/l), moderate-level (20.00–49.99 mg/l) and low-level (0.00–19.99 mg/l) of IAA production was 9 (7.09%), 23 (18.11%) and 95 (74.80%), respectively. The *B. cereus* group sp. strain SB127 produced IAA at the highest level (64.39 ± 3.94 mg/l), followed by the *B. subtilis* strain RD198 (61.58 ± 4.08 mg/l) and the *Pseudomonas* sp. strain LC182 (57.15 ± 4.61 mg/l).

**Table 2:**
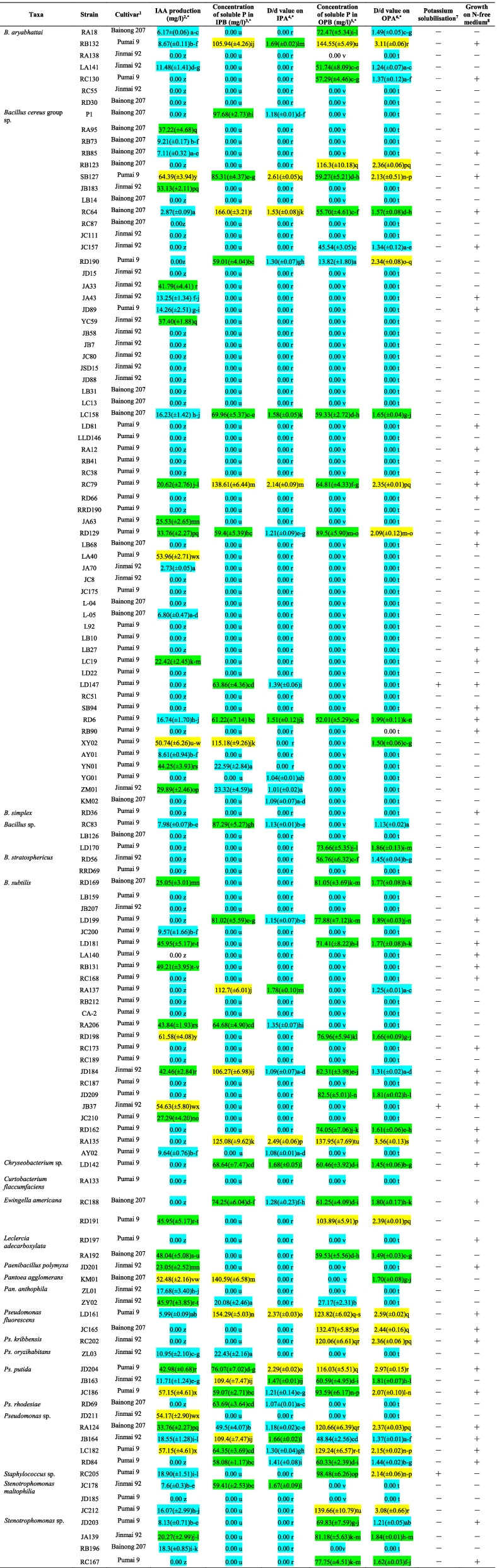
Biochemical properties of the endophytic bacterial strains isolated from three wheat cultivars

^1^ The winter wheat cultivars Jinmai 92, Bainong 207 and Pumai 9 were sampled from Yuncheng, Zhoukou and Nanyang cities, respectively, for the isolation of endophytic bacteria

^2^ Yellow, green, and blue shadows distinguish high- (≥ 50 mg/l), moderate- (20.00–49.99 mg/l) and low-levels (0.00–19.99 mg/l), respectively, of IAA production

^3^ Yellow, green, and blue shadows distinguish high- (≥ 100 mg/l), moderate- (50.00–99.99 mg/l) and low-levels (0–49.99 mg/l), respectively, of phosphate solubilising activity in IPB (inorganic phosphate broth)

^4^ Yellow, green, and blue shadows distinguish high- (D/d ≥ 2.00), moderate- (1.50 ≤ D/d < 2.00) and low-levels (D/d < 1.50), respectively, of phosphate solubilising activity in IPA (inorganic phosphate agar). D/d value = total diameter (colony + halo zone)/colony diameter (Kumar and Narula, 1999)

^5^ Yellow, green, and blue shadows distinguish high- (≥ 100 mg/l), moderate- (50.00–99.99 mg/l) and low-levels (0–49.99 mg/l), respectively, of phosphorus solubilising activity in OPB (organic phosphorus broth)

^6^ Yellow, green, and blue shadows distinguish high- (D/d ≥ 2.00), moderate- (1.50 ≤ D/d < 2.00) and low-levels (D/d < 1.50 mg/l), respectively, of phosphorus solubilising activity in OPA (organic phosphorus agar)

7,8 ′ + ′ represents positive reactions or growth; ′-′ represents negative reactions or no growth

* Different lowercase letters in the same column indicate significant differences at P ≤ 0.05 (ANOVA with Duncan’s post-test)

### Inorganic/organic phosphorus solubilisation by endophytic bacterial strains

The ability of solubilising inorganic/organic phosphorus was variable among 127 endophytic bacterial strains tested (Table 2). A total of 34 and 32 strains showed inorganic phosphorus-solubilising activities in IPB (27.77%) and IPA (25.20%) media, respectively. Twenty-nine strains showed inorganic phosphorus-solubilising activities in both IPB and IPA media therein. Based on a D/d value on IPA, the number of strains with a high-level (D/d ≥ 2.00), moderate-level (1.50 ≤ D/d < 2.00) and low-level (D/d < 1.50) phosphate-solubilising activity was 6 (4.72%), 8 (6.30%) and 113 (88.98%), respectively. Based on the concentration of available phosphorus in IPB, the number of strains with a high-level (≥ 100 mg/l), moderate-level (50–99.99 mg/l) and low-level (0–49.99 mg/l) inorganic phosphate-solubilising activity was 11 (8.66%), 18 (14.17%) and 98 (77.17%), respectively. On the other hand, a total of 43 and 46 strains showed organic phosphorus-solubilising activities in organic phosphorus broth (IPB) (33.86%) and organic phosphorus agar (IPA) (36.22%), respectively. Forty-two strains showed organic phosphorus-solubilising activities in both OPB and OPA media therein. Based on a D/d value on OPA, the number of strains with a high-level (D/d ≥ 2.00), moderate-level (1.50 ≤ D/d < 2.00) and low-level (D/d < 1.50) phosphate-solubilising activity was 17 (13.39%), 17 (13.39%) and 93 (73.23%), respectively. Based on the concentration of available phosphorus in OPB, the number of strains with a high-level (≥ 100 mg/l), moderate-level (50.00–99.99 mg/l) and low-level (0–49.99 mg/l) organic phosphorus-solubilising activity was 11 (8.66%), 28 (22.05%) and 88 (69.29%), respectively.

### Potassium-solubilising activity and growth on a nitrogen-free medium of endophytic bacterial strains

Only three of the 127 isolated strains showed a potassium-solubilising activity, expressed as the ability to produce halo zones in silicate-agar plates (Table 2). The *B. cereus* group sp. LD147, *B. subtilis* JB37 and *Staphylococcus* sp. RC205 created halo zones with diameters of 1.78 ± 0.06 mm, 1.26 ± 0.13 mm and 2.74 ± 0.15 mm, respectively. On the contrary, 48 (37.8%) of the 127 strains –from the taxa *B. cereus* group sp. (JA43, JC157, JD89, LB27, LB68, LC19, LD81, LD147, RA12, RB85, RB90, RC38, RC64, RC79, RD6, RD66, RD129, SB94, SB127), *B. aryabhattai* (RB132, RC130), *B. simplex* (RD36), *B. subtilis* (JB37, JD184, LA140, LD181, LD199, RA135, RB131, RC168, RC173, RC187, RD162), *Chryseobacterium* sp. (LD142), *L. adecarboxylata* (RD197), *Ew. americana* (RC188), *Pa. polymyxa* (JD201), *Ps. fluorescens* (JC165, LD161), *Ps. kribbensis* (RC202), *Ps. putida* (JC186, JD204), *Pseudomonas* sp. (JB164, LC182, RA124, RD84) and *Stenotrophomonas* sp. (JD203, RC167)– showed a positive growth response on nitrogen (N)-free plates (Table 2), indicating possible utilisation of atmogenic nitrogen by these bacterial endophytes.

### Efficacies of isolated endophytic bacteria in promoting wheat growth

**Fig. 4:**
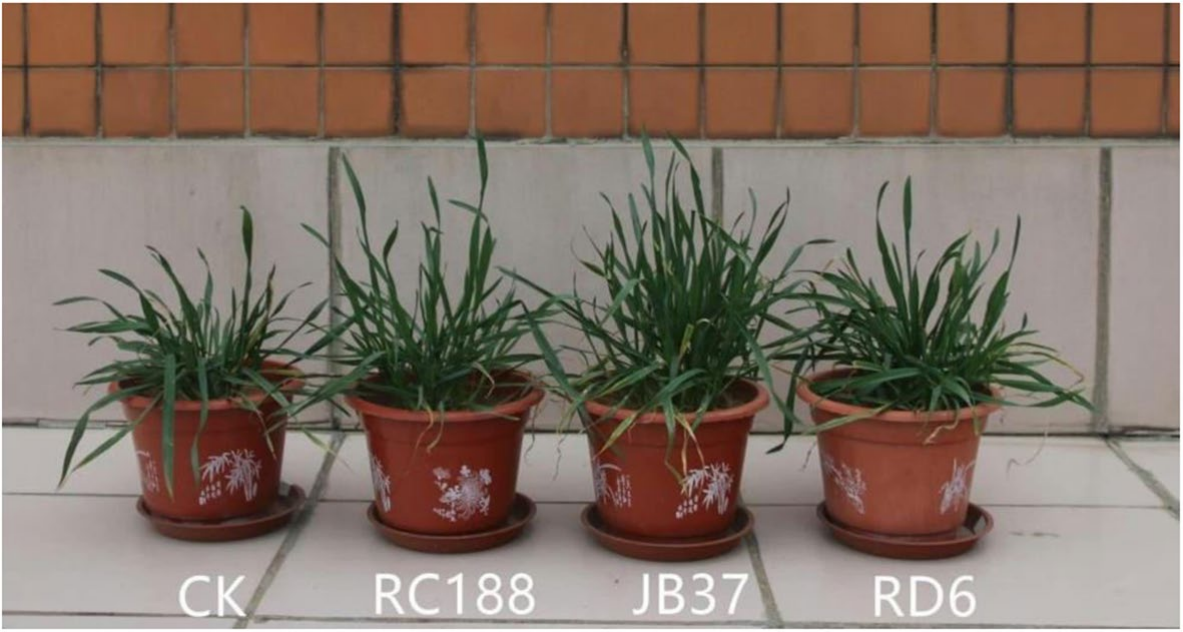
Representative photograph of the biofertiliser effect on wheat plants of our growth-promoting strains. From the right to the left are *Bacillus cereus* group sp. strain RD6, *B. subtilis* strain JB37, *Ewingella americana* strain RC188, and CK (uninoculated control). The photograph by Fahu Pang

**Fig. 5:**
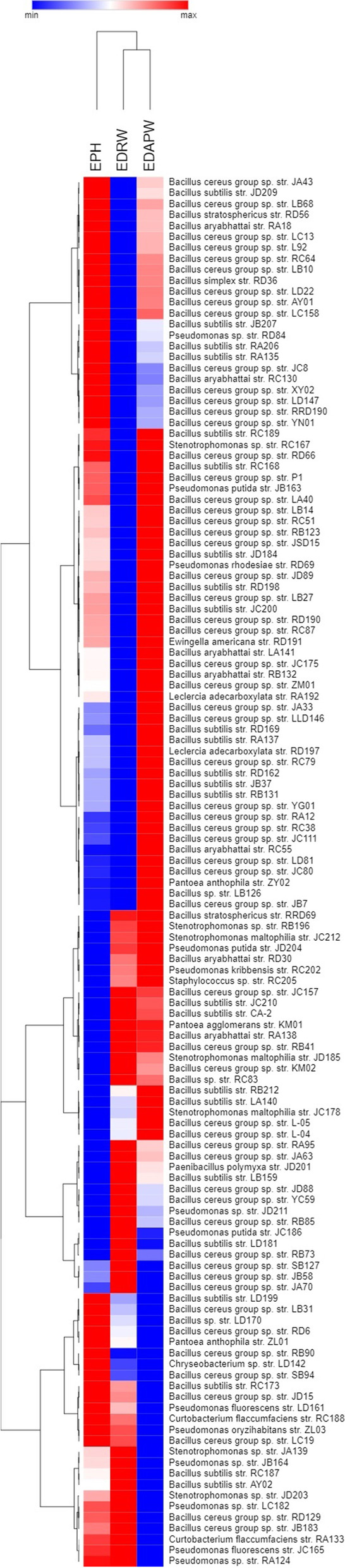
Hierarchical clustering of 127 isolated wheat bacterial endophytes regarding their growth-promoting effects. A cluster heatmap to visualise the saturation of colours indicating the associations among growth parameters (EPH: enhanced plant height, EDRW: enhanced dry root weight, EDAPW: enhanced dry above-part weight) -determined in pots trials- on the columns and the 127 wheat bacterial endophytes on the rows. The colour gradient represents the size of the data value; the closer the colour is to red, the higher is the data value. The data were clustered by Ward’s clustering with the Euclidean distance algorithm for similarity measure. For the analysis, Cluster 3.0 (http://bonsai.hgc.jp/~mdehoon/software/cluster/software.htm) and the Java Treeview software (http://jtreeview.sourceforge.net) were used

The growth of endophyte-inoculated wheat seedlings was compared with uninoculated (control) wheat seedlings in terms of enhanced plant height (EPH), enhanced dry root weight (EDRW) and enhanced dry above-ground part weight (EDAPW) on day 35 after sowing in soil with N-P-K macronutrients at low or suboptimal rates (Table S1, Fig. 4). The results were visualised in a clustered heatmap (Fig. 5); the dendrograms along the sides showed how the growth parameters (EPH, EDRW and EDAPW) and the 127 strains were independently clustered. In the clustered heatmap, EDAPW and EDRW showed more correlation than EPH. Out of the 127 strains, 59 (46.4%) showed the highest enhancement on EDAPW, 40 (31.4%) on EPH and 35 (27.5%) on ERDW. Growth inhibition (a negative value) was produced by 38 strains on ERDW, 15 on EDAPW and eight on EPH.

## Discussion

This study has analysed the diversity of culturable endophytic bacteria among all plant organs and during the different growth stages of three commercial winter wheat cultivars and their plant-growth-promoting traits. We sampled healthy plants since they host a more diverse population of endophytic bacteria than unhealthy plants [37]. We categorised 127 wheat endophytic strains randomly selected from 610 endophytic bacterial cultures in sixteen specified and six unspecified taxa, based on 16S rRNA gene sequence similarity with previously characterised bacterial species in the GenBank database. The 73 strains that resulted in unspecified taxa were included in the genera *Bacillus, Chryseobacterium, Pseudomonas, Staphylococcus* and *Stenotrophomonas*, in which *B. cereus* group sp. consisted of 59 (80.8%) unspecified strains. The sequence of the 16S rRNA gene has been widely used for molecular identification during extensive screening studies of beneficial bacteria from the environment. However, its resolution to identify a few genetically close species (e.g. *B. cereus*, *B. thuringiensis* and *B. anthracis*) is still far from clear. Indeed, *B. cereus*, *B. thuringiensis* and *B. anthracis* were considered a single species based on genetic evidence and classified into the *B. cereus* group [38]. In future, more specific identification of those strains with potential application in agriculture will involve other housekeeping genes such as *rpo*D [30], *gyr*A [39] and *gyr*B [40] that have proven helpful in discriminating closely related bacterial species.

We isolated endophytic bacteria from wheat using a robust method previously reported by our group [30]. Using microbial culturing methods, we showed that the surface sterilisation procedure effectively removed all surface-adhering microorganisms and that the obtained bacteria could be considered true endophytes. We found that the diversity of endophytic bacteria in wheat differed among plant organs, reflecting their adaptations and a different relationship with the host. The organ-specific and growth-stage-specific colonisation by some wheat endophytic bacteria might suggest a significant influence of plants' physiological status on bacterial growth and reproduction. In our study, the most diverse communities were residing within the wheat root. The plant root is more stable than above-ground tissues regarding environmental temperature and humidity variations and hence a preferred niche [34]. Some authors have suggested the endophyte microbiome as a subpopulation of the bacteria inhabiting the plant rhizosphere [24, 41]. Jin et al. (2014) reported that bacterial profiles obtained from the rhizosphere and roots of *Stellera chamaejasme* were similar [24], implying that bacteria residing in the rhizosphere might potentially enter and colonise the plant root from which the endophytic bacteria could expand toward plant above-ground tissues. On the other hand, endophytic bacteria can also use stomata in aerial parts (leaf and young stem) to enter the plant [42]. In tobacco plants, Ma and Xiao (2004) reported that endophytic bacteria were relatively more abundant in growing tissues [43]. Ongoing experiments in our laboratory with some strains showing good PGP performance tagged with the enhanced green fluorescent protein will determine their distribution within the wheat plant.

Out of the 51 strains isolated from the wheat root, more than 70% belonged to the Bacillus genus (*B. aryabhattai*, *B. cereus* group sp, *B. simplex*, *Bacillus* sp., *B. stratosphericus* and *B. subtilis*). Many *Bacillus* taxa have previously been described as endophytes [29, 44, 45]. In banana (*Musa* spp.), the highest number of endophytic isolates from plant root was also from the genus *Bacillus* [46]. However, the predominance of members of Firmicutes among the endophyte communities is not frequent; commonly, the Proteobacteria is reported as the most represented phylum in several plant species of agronomic interest, including wheat [20, 33, 47]. Each plant endophyte population can be significantly influenced by multiple biotic and abiotic factors [48]; for that reason, comparisons between plant endophyte communities are complex. Therefore, culture-based or not, broad-screening approaches are necessary to understand the ecology behind the plant-endophyte association to develop more effective biocontrol and host-specific plant-growth-promoting agents.

We isolated 11 strains of five taxa (*B. cereus* group sp, *B. subtilis*, *Pan. agglomerans*, *Pan. anthophila* and *Ps. oryzihabitans*) from wheat seeds of three Chinese commercial cultivars (Pumai 9, Bainong 207 and Jinmai 92). Dias Herrera et al. (2016) reported the obtainment of six isolates of three different genera (Paenibacillus, Bacillus, Pantoea) from a commercial wheat variety widely sown in Argentina [33]. In general, *Bacillus* and *Pseudomonas* have been the most frequent genera found in plant seeds, though *Paenibacillus*, *Micrococcus*, *Staphylococcus*, *Pantoea* and *Acinetobacter* can also be isolated [49]. Usually, the low number of bacterial genera recovered from the reproductive organ has been attributed to the specificity of seed habitat and limitations of culture-dependent techniques [49]. However, other factors such as seed quality and conditions and duration of seed storage may also affect the type and number of endophytic bacteria isolated from this plant organ [50–52].

The plant growth stage is another factor that affects the diversity and multiplication of endophytic bacteria within the host [53]. Our study is the first to report the variety of endophytic bacteria at the four growth stages (tillering, jointing, heading, and seed-filling) on the three winter wheat cultivars (Pumai 9, Bainong 207 and Jinmai 92). We found the isolation frequencies of most taxa increased from tillering to seed-filling. The three taxa (*B. aryabhattai*, *B. cereus* group sp. and *B. subtilis*) were isolated from all wheat growth stages, with the *B. cereus* group sp. predominating significantly over the rest of taxa. The ten taxa (*B. simplex*, *Chryseobacterium* sp., *E. americana*, *Pae. polymyxa*, *Pan. agglomerans*, *Pan. anthophila*, *Ps. oryzihabitans*, *Ps. rhodesiae*, *Stathylocouuss* sp. and *Ste. maltophilia*) were detected only in the mature plant (after jointing or heading stage). Similarly, Robinson et al. (2016) showed a slight increase in the total abundance of wheat endophytes in a second of two points of sampling (May and July) [34]. In maize, the highest population of endophytic bacteria in root, stem and leaf organs were isolated at the flowering, followed by vegetative and maturity growth stages [54]. Yu et al. (2015) have suggested changes in nutrients' availability, leaf size, and metabolites contents in sweet leaf (*Stevia rebaudiana* L.) could be the reason for dynamic changes of endophytic bacterial communities throughout plant growth [55]. However, Redford and Fierer (2009) have reported a reduction in endophyte number through plant growth [56].

The number of isolated bacterial taxa at the different growth stages was 9, 7, 10 and 16 for tillering, jointing, heading and seed-filling. The reason for a higher number of isolated taxa at the seed-filling stage could be more likely associated with environmental factors. For example, the tillering, jointing and heading of winter wheat plants commonly occur from November to late April when the temperatures are still colder than during the seed-filling stage, predominantly occurring in May. The cold temperatures decrease plant metabolism and could affect the multiplication of endophytic bacteria. From the heading stage, the number of isolated bacterial taxa increased, probably associated with more availability of photosynthetic products as the temperature rises and plants receive more sunlight. During the seed-filling stage, cuticular permeability might increase in all plant organs, possibly due to ageing [57], resulting in more microbes entering a wheat plant; consequently, the maximal number of bacterial taxa obtained at this particular growth stage.

Interestingly, our study did not detect the endophytic species *Brevibacillus borstelensis* and *B. agri*, previously described in wheat [58]. It is reasonable to speculate that the wheat genotype might influence the host plant's acceptability of specific bacterial species as an endophyte. On the other hand, endophytes *B. aryabhattai*, *B. stratosphericus*, *L. adecarboxylata*, and *Ps. oryzihabitans* were previously isolated from plants other than wheat [59–63]; therefore, our report is the first on the four taxa as wheat endophytes.

The replication of microbes in the endosphere of healthy plants suggests the host could benefit from harbouring them. Endophytes have been found to promote plant growth by producing phytohormones, solubilising phosphate and potassium, and fixing nitrogen from the air [64]. Some endophytic strains of *Bacillus*, *Chryseobacterium*, *Pseudomonas*, *Staphylococcus* and *Stenotrophomonas* secrete plant hormones such as IAA [64–68]. We performed qualitative and quantitative analyses of IAA production to the totality of tested wheat endophytic bacterial strains. Of the 127 strains, 58 (45.67%) could produce IAA, belonging to *B. aryabhattai*, *B. cereus* group sp., *Bacillus* sp., *B. subtilis*, *E. americana*, *L. adecarboxylata*, *Pae. polymyxa*, *Pan. agglomerans*, *Pan. anthophila*, *Ps. fluorescens*, *Ps. oryzihabitans*, *Ps. putida*, *Pseudomonas* sp., *Staphylococcus* sp., and *St. maltophilia*. All isolated *Pae. polymyxa*, *Pan. agglomerans*, *Pan. anthophila*, *Ps. oryzihabitans*, *Ps. putida*, and *Staphylococcus* sp. strains produced IAA in vitro; however, the number of strains analysed in this study was insufficient to fully support IAA production as a species-specific trait for a symbiotic relationship with their host plants. Egorshina et al. (2012) found that wheat seeds treated with *B. subtilis* spores transiently increased IAA concentrations in roots and shoots of seedlings [68]. In *Vigna radiata* L., the production of IAA by plant-associated *Bacillus* sp. was reported to correlate significantly with the shoot length, pod number and grain weight [67]. On the other hand, the IAA is a secondary metabolite, and its production by a bacterial strain/isolate is unstable and easily influenced by cultural conditions. For this reason, although more than 50% of our tested endophytic bacterial strains showed no IAA activity in vitro, it does not negate the possibility that they can produce IAA in vivo due to changes in nutritional conditions. Indeed, Shi et al. (2009) reported IAA production by endophytic bacterial isolates (including those that showed no IAA activity in glucose-peptone broth) could be significantly enhanced in a medium supplemented with L-tryptophan [65].

We assayed the ability to solubilise phosphate for plant nutrition of the 127 wheat endophytic strains from inorganic [TCP, Ca_3_(PO_4_)_2_] and organic (lecithin) phosphorous sources, using both qualitative (phosphate-agar) and quantitative (phosphate-broth) assays. For some endophytic strains, we found inconsistencies in phosphate-solubilising activities between phosphate-agar and phosphate-broth assays -which were more prominent with the inorganic source- that we attributed to a not homogeneous distribution of the insoluble phosphorus in the solid agar plates. Previously, Nautiyal Shekar (1999) recommended the combined use of phosphate-agar and phosphate-broth assays for the reliable isolation of phosphate-solubilising bacteria (PSB) [69]. Though the TCP is the most frequently used method to isolate and test PSB, Bashan et al. (2013) have suggested combining two or three metal-P compounds instead as a more reliable approach to define the isolates as true PSB [70].

We found potassium solubilising bacteria poorly represented in our wheat endophytes with only one strain from each *B. cereus* group sp. LD147, *B. subtilis* JB37 and *Staphylococcus* sp. RC205. In contrast, 48 strains from *B. aryabhattai*, *B. cereus* group sp., *B. simplex*, *B. subtilis*, *Chryseobacterium* sp., *E. americana*, *L. adecarboxylata*, *Pae. polymyxa*, *Ps. fluorescens*, *Ps. kribbensis*, *Ps. putida*, *Pseudomonas* sp. and *Stenotrophomonas* sp. grew on the N-free medium. Interestingly, 33 of the 48 potential nitrogen-fixer strains (69%) belonged to the genus Bacillus, previously described as such by Raymond et al. [71]. Nitrogen-fixing endophytes provide nitrogen to host plants. Further experiments on best candidate strains will search for the expression of the *nif*H gene that encodes the Fe-nitrogenase subunit of the nitrogenase complex and is strong evidence of the nitrogen-fixing ability in bacteria [72].

Pot trials revealed several PGP bacterial endophytes with great potential as biofertilisers of winter wheat plants. We found 19 strains enhancing all of the tested PGP parameters by 20% or greater and were classified as complete PGP (c-PGP) strains with a high- or moderate-level of PGP activity (Table S1). Nine c-PGP strains (*B. cereus* group sp. strains RB73, RC64, JA43, YC59 and L-05; *Pan. agglomerans* strain KM01; *Pan. anthophila* strain ZY02; *Ps. putida* strain JD204; *Pseudomonas* sp. strain JD211) produced IAA; seven (*B. cereus* group sp. strains RC64 and KM02; *Bacillus* sp. LD170; *Chryseobacterium* sp. strain LD142; *Pan. agglomerans* strain KM01; *Pan. anthophila* strain ZY02; *Ps. putida* strain JD204) solubilised phosphorus; six (*B. cereus* group sp. strains RC64, JA43 and LD81; *B. subtilis* strain LA140; *Chryseobacterium* sp. strain LD142; *Ps. putida* strain JD204) grew on N-free medium. Five of the 19 c-PGP strains (*B. aryabhattai* strain RD30; *B. cereus* group sp. strains JC111, JB7 and L-04; *Bacillus* sp. strain LB126) were negative for these three growth-promoting traits during in vitro assays. The existence of c-PGP strains lacking IAA production, phosphorus solubilisation and growth on N-free medium has yet to be confirmed in vivo, but it also could suggest another factor(s) may be involved in their PGP effects on wheat plants. Finally, ten strains produced growth data values below 10% for the three growth parameters and were referred to as non-growth promoting strains (i.e. *B. cereus* group sp. strains RB85, LB14, JA33, LC13 and LB68, *B. subtilis* strains JB207, LD199 and JD209, and *Stenotrophomonas* sp. strain JD203).

Further identification of growth-promoting factor(s) other than IAA production, phosphorus solubilisation and growth on N-free medium is needed for a complete understanding of wheat growth promotion by our endophytic bacteria and adequate characterisation of those with potential as biofertilisers. The contribution to plant growth differed among taxa, possibly influenced by the ecological niche of endophytes, their displayed plant-growth-promotion features, and the specific plant-endophyte interaction. Previously, we showed bacterial endophytes’ ability to produce IAA and/or solubilise organic/inorganic phosphorous significantly positively correlated with wheat growth promotion [73]. The preliminary analysis of ongoing field trials with some of the strains that performed better in the pot trial has confirmed the plant-growth-promoting nature of the endophytes but also showed the enhancement of some host resistance-related enzymes (phenylalanine ammonia-lyase, polyphenol oxidase, peroxidase, catalase and superoxide dismutase) and higher levels of proline and flavonoids in treated relative to uninoculated plants (unpublished results).

## Conclusions

In summary, we have characterised the diversity of PGP endophytic bacteria in three winter wheat cultivars grown in three different locations in China and found it to be influenced by the plant organ and growth stage. Root emerged as the main reservoir and preferred organ for screening PGP endophytic bacteria in this crop. Many of the tested endophytic bacterial strains could produce IAA, solubilise phosphate, grow on an N-free medium and promote wheat growth in pot trials in soil with N-P-K macronutrients at low or suboptimal rates. The results have revealed several PGP strains that could be used as biofertilisers for the sustainable production of winter wheat varieties in China.

## Methods

### Wheat cultivars

This study used a total of three commercial winter wheat cultivars, including Pumai 9 (registration no. 2005012), Bainong 207 (registration no. 2013010) and Jinmai 92 (registration no. 2012012), which have been approved by the Ministry of Agriculture and Rural Affairs of the People’s Republic of China and are widely grown in traditional farming systems in both Henan and Shanxi provinces. Pumai 9, Bainong 207 and Jinmai 92 seeds are maintained and marketed by the Puyang Academy of Agricultural Sciences (Henan, China), the Huaguan Seed Technology Co. Ltd (Henan, China) and the Shanxi Academy of Agricultural Sciences (Shanxi, China), respectively.

### Sampling sites

The study took place from December 2012 to May 2013. The sampling sites were located in private lands in Nanyang (33° 01'N, 112° 29'E) and Zhoukou (33° 38'N, 114° 38'E) cities of Henan province and Yuncheng city (35° 02'N, 111° 01'E) of Shanxi province, in China. In all cases, the landowners permitted sampling. In sampling areas, the seasons for sowing seeds were from mid-October to late October and harvesting between late May to early June of next year, based on cultivars used and weather conditions. While farmers in Henan province grow wheat in 'yellow cinnamon', 'yellow–brown' and 'coarse-bone' soil types, Shanxi's soil types for wheat production are 'cinnamon' or 'coarse-bone', according to the Chinese Soil Genesis Classification System [74]. These soil types belong to the Argosol order in the Chinese soil taxonomy, with planosols/albeluvisols/alisols/luvisols as the most similar soil types in the world reference base for soil resources (https://www.fao.org/3/W8594E/W8594E00.htm). The climatological mean values (daily mean temperature and precipitation) from December to May for Henan and Shanxi provinces were 13 °C and 33 mm and 10.6 °C and 20.2 mm, respectively (China weather; https://www.weather-forecast.com/).

The fertilisation scheme of winter wheat cultivation involved two major fertiliser applications. The first application (basal dressing) was carried out before the rotary tillage of soil in preparation for seed sowing (early in October) by adding 750 kg/ha of compound fertiliser (17% nitrogen, 17% P_2_O_5_, and 17% K_2_O). The second application (top dressing) took place at the jointing stage (March) and included 120 kg/ha of urea (46% nitrogen). For the control of wheat aphids, imidacloprid 10% wettable powder (112.5 g/ha) was sprayed early in April. Omethoate 40% emulsifiable concentrate (1500 ml/ha) was sprayed in mid-April to control red mites.

### Sample collection

Healthy winter wheat plants were sampled separately at tillering (10 weeks after sowing), jointing (23 weeks after sowing), heading (27 weeks after sowing), and seed-filling (30 weeks after sowing) stages from five sampling points (20 plants per point) per field. The samples of cultivars Pumai 9, Bainong 207 and Jinmai 92 were collected from Nanyang, Zhoukou and Yuncheng, respectively. The wheat samples of the same cultivars were separately pooled and used for the isolation of bacterial endophytes soon after collection or preserved at 4 °C for 1–2 days before the analysis.

### Isolation of endophytic bacteria

Wheat endophytic bacteria were isolated according to Pang et al. [30]. Leaf, stem, root and seed samples (1.5 g each) were washed with abundant tap water and cut out into small pieces (ca. 5 mm long) before being treated with 75% ethanol for 30 s and 0.1% mercuric chloride (2–3 min for the leaf, 3–4 min for the stem, 5 min for the root, and 8 min for the seed) for surface disinfection. Then, treated samples were rinsed with sterile water at least five times and used to prepare tissue suspensions in sterile phosphate-buffered saline (pH 7.0) with a sterile mortar. The surface sterilisation was verified by spreading tissue suspension (10^–3^ to 10^–6^ dilutions) and rinsing water samples on nutrient agar (NA; 3 g of beef extract, 5 g of peptone, 5 g of sodium chloride, and 15 g of agar in 1000 ml of water) and incubating the NA plates at 28 °C for 5–7 days [75, 76]. The process was successful if bacterial colonies were obtained only on NA plates containing tissue suspension but not rinsing water. Thus, bacterial colonies from tissue suspensions prepared from surface-sterilised samples were designed as wheat endophytes. The endophytic bacteria were further purified by a single-colony isolation approach (repeated three times), and strain replicates were stored at 4 °C onto NA slants and at -70 °C as glycerol stocks.

### Identification of endophytic bacteria

The identification of endophytic bacteria was performed based on 16S rRNA gene sequences, as previously described [29]. The strains were inoculated in NB medium and incubated at 28 °C with shaking (180 rpm) for 24 h before genomic DNA extraction. Sequences of the 16S rRNA gene (~ 1500 base pairs) were PCR-amplified using universal primers 27F (forward) 5'-AGAGTTTGATCATGGCTCAG-3' and 1492R (reverse) 5'-GGTACCTTGTTACGACTT-3' [77]. PCR products were then visualised by electrophoresis, purified using a kit from TransGen Biotech Co., LTD (Beijing) and sent to Synbio Technologies (Suzhou) for DNA sequencing. Homologous DNA sequences retrieval and analysis were conducted using the BLAST algorithm (www.blast.ncbi.nlm.nih.gov/Blast.cgi). For the assignment of taxonomic affiliation at the species level, a per cent identity of 16S rRNA gene sequence above 99% with a specific bacterial species was enough to give a tentative scientific name to the strain. In contrast, a per cent identity of the sequence above 99% with two or more bacterial species or lower than 99% represented an unidentified species named the genus plus "sp". The final identification of the bacterial strains was based on a neighbour-joining phylogenetic tree of bacterial 16S rRNA gene sequences established with the software MEGA version 4.0. The diversity (Shannon and Simpson indexes) and species evenness (Evenness index) estimates per plant organ or per cultivar were calculated online (https://www.alyoung.com/labs/biodiversity_calculator.html).

### Detection of IAA-producing strains and estimation of IAA

To detect IAA-producing endophytic bacteria, each strain was inoculated into 50 ml of yeast mannitol broth (YMB) containing 0.1 g tryptophan, 0.1 g NaCl, 0.2 g MgSO_4_, 0.5 g KH_2_PO_4_, 1.0 g yeast extract and 10 g mannitol in 1000 ml distilled water (pH 6.8–7.2) and incubated at 28 °C for 3–5 d at 160 rpm. A volume of 100 µl from each bacterial suspension (10^7^ CFU/ml) was dropped on a well of a standard colourimetric plate and mixed with an equal amount of the Salkowski colourimetric reagent (a 100 ml solution consisted of 98 ml 35% HClO_4_ + 2 ml 0.5 mol/l FeCl_3_) [78] at room temperature for 30 min. YMB (without bacteria) with the Salkowski colourimetric reagent represented the negative control, whereas the Salkowski colourimetric reagent with IAA (50 mg/l) represented the positive control. When the strain produced IAA, the solution turned pink, and the reaction was considered positive. The intensity of the pink colour correlates with the amounts of IAA produced. On the contrary, no change in the colour of the solution was observed in the absence of IAA production. The IAA levels produced by IAA-positive strains were estimated colourimetrically by Salkowski colourimetric reagent following the method described by Gordon and Weber [79].

### Qualitative and quantitative determination of phosphate-solubilising activity

The bacterial strains were grown on inorganic phosphorus agar (IPA) and organic phosphorus agar (OPA) media to evaluate their phosphate-solubilising activity qualitatively. The IPA medium consisted of 10 g glucose, 0.5 g (NH_4_)_2_SO_4_, 5 g Ca_3_(PO_4_)_2_, 0.3 g NaCl, 0.03 g FeSO_4_·7H_2_O, 0.3 g KCl, 0.03 g MnSO_4_·4H_2_O, 0.4 g yeast extract, and 15 g agar in 1000 ml distilled water (pH 7.0–7.5), whereas, the OPA medium consisted of 10 g glucose, 0.5 g (NH_4_)_2_SO_4_, 0.3 g K_2_SO_4_, 0.3 g NaCl, 0.03 g MnSO_4_·4H_2_O, 0.03 g FeSO_4_·7H_2_O, 0.2 g lecithin, 5 g CaCO_3_, 0.4 g yeast extract and 15 g agar in 1000 ml distilled water (pH 7.0–7.2). The strains were separately point-inoculated on IPA and OPA plates (5 plates per strain, 3 locations per plate) and incubated at 28 °C for 3–5 d. A halo formed around the bacterial colony indicated phosphorus solubilisation. The diameter of the halo zone produced by bacterial colonies of each strain was determined with a vernier calliper (CD-S15M; Mitutoyo Corporation, Japan). The strain's ability to solubilise phosphorus on IPA/OPA media was evaluated by its D/d value on the media, where D = total diameter (colony + halo zone) and d = colony diameter [80].

Bacterial strains with phosphate-solubilising activity on IPA and/or OPA plates were grown separately in inorganic phosphorus broth (IPB) and organic phosphorus broth (OPB) to further estimate the production of available phosphorus quantificationally. The IPB and OPB were the same as IPA and OPA, respectively, except for the agar. A volume of 1 ml from each bacterial suspension (10^7^ CFU/ml) was added to 50 ml of IPB or OPB in a 250-ml Erlenmeyer flask. As a control, an equal amount of sterile water was added to the medium to replace the bacterial suspension. The bacterial cultures were incubated at 28 °C for 3–7 d under 160 rpm and then centrifuged at 10,000 rpm for 10 min. The supernatants were used for the quantitative determination of available phosphorus contents by the Mo-Sb colourimetric method [81]. Each strain was replicated three times.

### Determination of potassium-solubilising activity

The bacterial strains were separately grown on a silicate medium (5 g sucrose, 0.5 g MgSO_4_, 2.0 g CaSO_4_, 0.4 g Na_2_HPO_4_, 0.005 g FeCl_3_, 1.0 g glass powder, 15 g agar in 1000 ml of distilled water; pH 6.8–7.2) at 28 °C for 48–72 h to evaluate their potassium-solubilising activity. A halo zone formed around the bacterial colony indicated potassium solubilisation [82]. The diameter of the halo zone produced by bacterial colonies of each strain was determined with a vernier calliper (CD-S15M; Mitutoyo Corporation, Japan). The strain's ability to solubilise potassium was evaluated by its D/d value on the silicate medium, where D = total diameter (colony + halo zone) and d = colony diameter.

### Bacterial growth on N-free medium

The ability of endophytic bacteria to grow on an N-free medium was analysed for possible utilisation of atmogenic nitrogen source. First, the bacterial strains were separately inoculated on NA plates to activate growth. Then, a loopful of cells from a single colony was picked and streaked on an N-free medium consisting of 0.2 g K_2_HPO_4_, 0.2 g MgSO_4_, 0.2 g NaCl, 2.0 g CaCO_3_, 10.0 g mannitol, 0.1 g CaSO_4_, and 15 g agar in 1000 ml water, and incubated at 28 °C for 48–72 h. The strain that grew well on the N-free medium for three rounds of successive sub-culturing was scored as a positive reaction indicating possible utilisation of atmogenic nitrogen [83].

### Pot trials

Each strain was grown in 50 ml of NB at 28 °C on a shaker (160 rpm) until the culture OD_600_ reached approximately 0.7–0.8, measured in a spectrophotometer model SP-752 (Shanghai Spectrum Instruments Co., Ltd.). Colony-forming units (CFU) were determined for all cultures by plating appropriate dilutions in NA plates. Bacterial cultures were assayed for growth promotion of Pumai 9, a wheat variety bred by the Puyang Academy of Agricultural Sciences (China, 2000). Pumai 9 is considered a high-yielding medium-maturity cultivar with lodging resistance, drought tolerance and moderate resistance to powdery mildew, leaf rust, leaf blight, stripe rust and sheath blight diseases.

For pot trials, Pumai 9 seeds came from a single batch. Seeds surface-disinfection consisted of a washed step with sterile distilled water for 5–10 min and then treated with 75% ethanol for 30 s, dipped in 0.1% mercuric chloride for 8 min and finally rinsed with sterile water at least five times. Seeds were soaked in each bacterial suspension (10^6^ CFU/ml) for 24 h and then spread uniformly on a sterilised filter paper moistened with sterile water in a 9-cm-diameter Petri dish. Wheat seeds treated with NB alone were used as the control. Endophyte-treated and control wheat seeds were incubated at 25 °C to accelerate germination. As seeds showed white sprouts, they were spot-sown at one-cm depth in pots (25 cm in up-diameter, 15 cm in bottom-diameter, and 20 cm in depth) filled with soil until about 2.5-cm space between the top of the soil and rim of the pot. The type of soil in pots was 'yellow cinnamon' containing 547.4 mg/kg, 16 mg/kg, and 53.5 mg/kg of total N, and available P and K, respectively, which were determined based on a routine method [84]. These macronutrients levels are considered insufficient for N and K and suboptimal for P [85]. Thirty seeds were sown per pot, but only 20 evenly grown seedlings were used per pot. The seedlings were grown for 35 days, from April 15 to May 20 in 2014, under natural conditions, watered with tap water twice a day (in the morning and the evening) as needed. Monthly average temperatures ranged between 7 °C and 29 °C. No symptoms of plant micronutrients deficiency were detected during the growing period. Growth parameters (i.e. plant height and dry weight) for each treatment were investigated as previously described [31].

### Statistical analysis

Data were analysed using the SPSS software vs. 16.0. First, data were tested for Normality. Then, a one-way analysis of variance (One-way ANOVA) in conjunction with post-hoc Duncan's multiple range test was performed to determine differences among treatments for diversity and species evenness indexes, IAA production and phosphorous solubilisation at *P* ≤ 0.05 as the significance level. In all cases, the experiments were carried out at least twice with three independent replicates, and similar results were obtained. Still, the data from one representative experiment are shown. The standard deviation of means was used to compare the replicates.

## Supplementary Information

**Additional file 1.**

## Data Availability

All data generated or analysed during this study are included in this published article. The 16S rRNA gene sequence data that support the findings of this study have been deposited in GenBank (http://www.ncbi.nlm.nih.gov) with the accession numbers [KF751619; KJ155727; KJ534397; KJ534398; KJ534399; KJ534400; KJ534401; KJ534402; KJ534403; KJ534404; KJ534405; KJ534406; KJ534407; KJ534408; KJ534409; KJ534410; KJ534411; KJ534412; KJ534413; KJ534414; KJ534415; KJ534416; KJ534417; KJ534418; KJ534419; KJ534420; KJ534421; KJ534422; KJ534423; KJ534424; KJ534425; KJ534426; KJ534427; KJ534428; KJ534429; KJ534430; KJ534431; KJ534432; KJ534433; KJ534434; KJ534435; KJ534436; KJ534437; KJ534438; KJ534439; KJ534440; KJ534441; KJ534442; KJ534443; KJ534444; KJ534445; KJ534446; KJ534447; KJ534448; KJ534449; KJ534450; KJ534451; KJ534452; KJ534453; KJ534454; KJ534455; KJ534456; KJ534457; KJ534458; KJ534459; KJ534460; KJ534461; KJ534462; KJ534463; KJ534464; KJ534465; KJ534466; KJ534467; KJ534468; KJ534469; KJ534470; KJ534471; KJ534472; KJ534473; KJ534474; KJ534475; KJ534476; KJ534477; KJ534478; KJ534479; KJ534480; KJ534481; KJ534482; KJ534483; KJ534484; KJ534485; KJ534486; KJ534487; KJ534488; KJ534489; KJ534491; KJ534492; KJ534493; KJ534494; KJ534495; KJ534496; KJ534497; KJ534498; KJ534499; KJ534450; KJ534501; KJ564121; KJ564122; KJ564123; KJ564124; KJ564125; KJ564126; KJ564127; KJ564128; KJ564129; KJ534490; KJ948663; KJ948664; KJ948665; KJ948666; KJ948667; KJ948668; KJ948669; KJ948670; KJ948671; KJ948672; KJ948673].

## Abbreviations

IAA: Indole-3-acetic acid
MSS: Maximal Similarity Score
IPA: Inorganic phosphorus agar
IPB: Inorganic phosphorus broth
OPA: Organic phosphorus agar
OPB: Organic phosphorus broth
GPS: Growth-promoting strains
PSB: Phosphate-solubilising bacteria
NA: Nutrient agar
NB: Nutrient broth
YMB: Yeast mannitol broth
CFU: Colony-forming units
N: Nitrogen
P: Phosphorus
K: Potassium
c-GPS: Complete growth promoting strains
IGPS: Incomplete growth promoting strains
One-way ANOVA: One-way analysis of variance
