# Supplementary material for: Plant organ- and growth stage-diversity of endophytic bacteria with potential as biofertilisers isolated from wheat (Triticum aestivum L.)
Source: BMC Plant Biol. 2022 Jun 6;22:276. doi: 10.1186/s12870-022-03615-8 (PMC9169407; doi:10.1186/s12870-022-03615-8)
Supplement: Supplementary file 1 — Additional file 1. [file 12870_2022_3615_MOESM1_ESM.docx]

**Plant organ- and growth stage-diversity of endophytic bacteria with potential as biofertilisers isolated from wheat (*Triticum aestivum* L.)**

Fahu Pang, Aili Tao, Camilo Ayra-Pardo, Tan Wang, Ziwei Yu, Siliang Huang

**Table S1. Growth promoting activities of the endophytic bacterial strains isolated from three wheat cultivars.**

| **Taxa** | **Strain** | **EPH^1^ (%)** | **EDRW^2^ (%)** | **EDAPW^3^ (%)** |
| --- | --- | --- | --- | --- |
| *B. aryabhattai* | RA18 | 22.6 | -31.3 | 2.5 |
|  | RB132 | 24.3 | 18.5 | 29.7 |
|  | RA138 | 14.1 | 40.6 | 39.0 |
|  | LA141 | 1.3 | -28.6 | 29.6 |
|  | RC130 | 26.4 | 15.2 | 18.0 |
|  | RC55 | 15.2 | 14.5 | 50.8 |
|  | RD30 | 20.5 | 34.5 | 38.7 |
| *Bacillus cereus* group sp*.* | P1 | 8.9 | -28.1 | 17.5 |
|  | RA95 | 12.3 | 33.2 | 24.8 |
|  | RB73 | 33.4 | 55.2 | 38.3 |
|  | RB85 | -7.2 | 7.9 | -1.4 |
|  | RB123 | 2.3 | -24.5 | 18.6 |
|  | SB127 | 22.1 | 35.0 | 17.7 |
|  | JB183 | 17.7 | 22.1 | 3.7 |
|  | LB14 | -29.3 | -48.8 | -16.5 |
|  | RC64 | 45.7 | 26.9 | 40.6 |
|  | RC87 | 24.0 | 12.4 | 29.7 |
|  | JC111 | 45.0 | 43.3 | 54.1 |
|  | JC157 | 7.5 | 11.0 | 10.6 |
|  | RD190 | 12.6 | -31.4 | 33.6 |
|  | JD15 | 27.7 | 21.9 | 6.6 |
|  | JA33 | -11.8 | -19.0 | 7.6 |
|  | JA43 | 54.0 | 26.3 | 43.0 |
|  | JD89 | 34.0 | 4.2 | 49.9 |
|  | YC59 | 31.8 | 55.4 | 41.8 |
|  | JB58 | 8.6 | 27.5 | 1.6 |
|  | JB7 | 29.1 | 28.3 | 44.1 |
|  | JC80 | 9.3 | 8.4 | 20.5 |
|  | JSD15 | -6.4 | -40.4 | 18.3 |
|  | JD88 | 8.2 | 39.5 | 21.4 |
|  | LB31 | 21.7 | 14.0 | 9.3 |
|  | LC13 | 4.4 | -43.7 | -12.5 |
|  | LC158 | 32.6 | 11.6 | 28.5 |
|  | LD81 | 24.5 | 22.6 | 53.4 |
|  | LLD146 | 1.4 | -14.4 | 41.0 |
|  | RA12 | 14.0 | 11.1 | 37.9 |
|  | RB41 | 8.5 | 52.5 | 49.6 |
|  | RC38 | 2.9 | -3.1 | 41.9 |
|  | RC79 | 32.5 | 19.7 | 53.9 |
|  | RD66 | 17.2 | -29.3 | 18.3 |
|  | RRD190 | 17.5 | 0.7 | 6.4 |
|  | JA63 | 2.7 | 53.2 | 34.2 |
|  | RD129 | 15.8 | 18.8 | 2.0 |
|  | LB68 | 2.0 | -27.9 | -7.6 |
|  | LA40 | 12.2 | -1.5 | 14.6 |
|  | JA70 | -6.4 | 42.5 | -13.9 |
|  | JC8 | 16.5 | -38.0 | -23.7 |
|  | JC175 | 11.1 | -3.7 | 25.0 |
|  | L-04 | 44.7 | 49.0 | 54.0 |
|  | L-05 | 31.0 | 41.7 | 53.9 |
|  | L92 | 28.7 | 17.9 | 24.9 |
|  | LB10 | 21.3 | -33.4 | 7.1 |
|  | LB27 | 8.8 | -9.7 | 17.1 |
|  | LC19 | 54.9 | 48.6 | 12.0 |
|  | LD22 | 26.5 | 3.2 | 20.8 |
|  | LD147 | 35.8 | 19.6 | 24.5 |
|  | RC51 | 12.3 | -22.6 | 35.8 |
|  | SB94 | 18.7 | -12.4 | -17.9 |
|  | RD6 | 22.1 | 6.7 | -7.2 |
|  | RB90 | 13.9 | -0.2 | -0.46 |
|  | XY02 | 32.0 | 3.0 | 12.0 |
|  | AY01 | 15.0 | 7.0 | 13.0 |
|  | YN01 | 21.0 | 18.0 | 19.0 |
|  | YG01 | 22.0 | 20.0 | 26.0 |
|  | ZM01 | 27.0 | 14.0 | 40.0 |
|  | KM02 | 24.0 | 58.0 | 48.0 |
| *B. simplex* | RD36 | 25.7 | 3.5 | 19.9 |
| *Bacillus* sp. | RC83 | 0.5 | 39.6 | 31.2 |
|  | LB126 | 31.0 | 30.5 | 41.5 |
|  | LD170 | 38.4 | 31.2 | 25.7 |
| *B. stratosphericus* | RD56 | 12.7 | -34.8 | -4.9 |
|  | RRD69 | 6.7 | 15.3 | 15.7 |
| *B. subtilis* | RD169 | 7.2 | 1.9 | 26.3 |
|  | LB159 | 14.5 | 61.3 | 39.9 |
|  | JB207 | -21.9 | -78.7 | -52.7 |
|  | LD199 | -7.3 | -40.2 | -55.7 |
|  | JC200 | 7.2 | -9.9 | 14.8 |
|  | LD181 | 2.0 | 55.9 | 3.2 |
|  | LA140 | 20.6 | 31.9 | 47.6 |
|  | RB131 | 9.6 | -9.2 | 46.8 |
|  | RC168 | 1.4 | -49.2 | 13.8 |
|  | RA137 | 12.5 | -12.7 | 53.9 |
|  | RB212 | 19.9 | 33.3 | 45.5 |
|  | CA-2 | 6.9 | 56.0 | 48.6 |
|  | RA206 | -13.0 | -63.8 | -43.9 |
|  | RD198 | 21.2 | -13.1 | 40.6 |
|  | RC173 | 16.3 | 12.7 | 4.3 |
|  | RC189 | 6.0 | -44.1 | 10.8 |
|  | JD184 | 13.9 | -30.1 | 46.6 |
|  | RC187 | 28.2 | 39.3 | 16.2 |
|  | JD209 | 8.5 | -38.9 | -12.0 |
|  | JB37 | 4.0 | -7.0 | 25.4 |
|  | JC210 | 3.1 | 16.5 | 14.2 |
|  | RD162 | 1.1 | -13.5 | 32.7 |
|  | RA135 | 31.8 | 6.4 | 16.9 |
|  | AY02 | 26.0 | 40.0 | 12.0 |
| *Chryseobacterium* sp*.* | LD142 | 37.6 | 28.3 | 26.9 |
| *Curtobacterium flaccumfaciens* | RA133 | 17.8 | 19.1 | 7.6 |
| *Ewingella americana* | RC188 | 24.8 | 22.6 | 15.0 |
|  | RD191 | 26.8 | 13.6 | 33.2 |
| *Leclercia adecarboxylata* | RD197 | 13.7 | -13.1 | 58.6 |
|  | RA192 | 22.9 | 14.3 | 30.2 |
| *Paenibacillus polymyxa* | JD201 | 18.7 | 52.9 | 37.8 |
| *Pantoea agglomerans* | KM01 | 21.0 | 48.0 | 47.0 |
| *Pan. anthophila* | ZL01 | 32.6 | 2.7 | -29.0 |
|  | ZY02 | 22.0 | 21.0 | 43.0 |
| *Pseudomonas fluorescens* | LD161 | 51.2 | 39.7 | 20.0 |
|  | JC165 | 17.5 | 18.3 | 9.2 |
| *Ps. kribbensis* | RC202 | 0.3 | 29.5 | 38.9 |
| *Ps. oryzihabitans* | ZL03 | 21.0 | 19. 0 | 8.0 |
| *Ps. putida* | JD204 | 31.0 | 51.6 | 54.2 |
|  | JB163 | 26.3 | 18.8 | 28.0 |
|  | JC186 | 19.8 | 33.0 | 20.6 |
| *Ps. rhodesiae* | RD69 | 8.3 | -29.9 | 35.1 |
| *Pseudomonas* sp. | JD211 | 32.9 | 50.4 | 39.1 |
|  | RA124 | 26.0 | 26.6 | 7.8 |
|  | JB164 | 22.8 | 30.1 | 13.0 |
|  | LC182 | 20.6 | 21.7 | 14.6 |
|  | RD84 | 23.0 | -33.5 | -8.1 |
| *Staphylococcus* sp. | RC205 | 3.6 | 42.8 | 56.5 |
| *Stenotrophomonas maltophilia* | JC178 | 10.2 | 23.5 | 42.7 |
|  | JD185 | 15.6 | 56.2 | 45.7 |
|  | JC212 | 18.8 | 40.3 | 43.8 |
| *Stenotrophomonas* sp. | JD203 | 7.5 | 8.4 | 5.7 |
|  | JA139 | 17.3 | 20.5 | 12.8 |
|  | RB196 | 10.6 | 20.3 | 21.7 |
|  | RC167 | -0.9 | -47.7 | 1.1 |

^1^ EPH denotes per cent of enhanced plant height. The yellow, green and blue shadows are used to distinguish high- (≥40%), moderate- (20.1-39.9%) and low- (≤20.0%) EPH, respectively.

^2^ EDRW denotes per cent of enhanced dry root weight. The yellow, green and blue shadows are used to distinguish high- (≥40%), moderate- (20.1-39.9%) and low- (≤20.0%) EDRW, respectively.

^3^ EDAPW denotes per cent enhanced dry above-ground part weight. The yellow, green and blue shadows are used to distinguish high- (≥40%), moderate- (20.1-39.9%) and low- (≤20.0%) EDAPW, respectively.
